# A Cholecystokinin B Receptor-Specific DNA Aptamer for Targeting Pancreatic Ductal Adenocarcinoma

**DOI:** 10.1089/nat.2016.0621

**Published:** 2017-02-01

**Authors:** Gary A. Clawson, Thomas Abraham, Weihua Pan, Xiaomeng Tang, Samuel S. Linton, Christopher O. McGovern, Welley S. Loc, Jill P. Smith, Peter J. Butler, Mark Kester, James H. Adair, Gail L. Matters

**Affiliations:** ^1^ Department of Pathology, Gittlen Cancer Research Laboratories, Pennsylvania State University College of Medicine, Hershey, Pennsylvania.; ^2^ Department of Neural and Behavioral Sciences and the Microscopy Imaging Facility, Pennsylvania State University College of Medicine, Hershey, Pennsylvania.; ^3^ Department of Chemistry, Pennsylvania State University, University Park, Pennsylvania.; ^4^ Department of Materials Science and Engineering, Pennsylvania State University, University Park, Pennsylvania.; ^5^ Department of Biochemistry and Molecular Biology, Pennsylvania State University College of Medicine, Hershey, Pennsylvania.; ^6^ Department of Medicine, Georgetown University, Washington, District of Columbia.; ^7^ Department of Bioengineering, Pennsylvania State University, University Park, Pennsylvania.; ^8^ Department of Pharmacology, University of Virginia, Charlottesville, Virginia.

**Keywords:** pancreatic cancer, DNA aptamer, targeted nanoparticles, tumor imaging

## Abstract

Pancreatic ductal adenocarcinomas (PDACs) constitutively express the G-protein-coupled cholecystokinin B receptor (*CCKBR*). In this study, we identified DNA aptamers (APs) that bind to the *CCKBR* and describe their characterization and targeting efficacy. Using dual SELEX selection against “exposed” *CCKBR* peptides and *CCKBR*-expressing PDAC cells, a pool of DNA APs was identified. Further downselection was based on predicted structures and properties, and we selected eight APs for initial characterizations. The APs bound specifically to the *CCKBR*, and we showed not only that they did not stimulate proliferation of PDAC cell lines but rather inhibited their proliferation. We chose one AP, termed AP1153, for further binding and localization studies. We found that AP1153 did not activate *CCKBR* signaling pathways, and three-dimensional Confocal microscopy showed that AP1153 was internalized by PDAC cells in a receptor-mediated manner. AP1153 showed a binding affinity of 15 pM. Bioconjugation of AP1153 to the surface of fluorescent NPs greatly facilitated delivery of NPs to PDAC tumors *in vivo*. The selectivity of this AP-targeted NP delivery system holds promise for enhanced early detection of PDAC lesions as well as improved chemotherapeutic treatments for PDAC patients.

## Introduction

By 2030, pancreatic ductal adenocarcinoma (PDAC) is predicted to be the second leading cause of cancer-related deaths in the United States [1]. Most PDAC patients are not candidates for surgery, and systemic chemotherapy shows little benefit [2]. The dense stroma and hypovascularity of PDAC tumors decrease the bioavailability of systemically delivered drugs and contribute to chemoresistance. Delivery of conventional chemotherapeutics is also hampered by rapid drug clearance, metabolic inactivation, and systemic toxicities [3]. By engineering delivery systems with tumor cell targeting agents, off-target drug effects can be minimized and the delivery of cargo constituents can be increased [4]. In this study, we have utilized amorphous calcium phosphosilicate nanoparticles (CPSNPs): they are biocompatible and biodegradable composite particles less than 100 nm in diameter, which are attractive candidates for bioimaging and therapeutic delivery applications. CPSNPs remain intact in the blood stream but dissolve in the low pH of endocytic vesicles, resulting in the intracellular release of their cargo [5].

Targeting of nanoparticle (NP) generally uses ligands, such as peptides or antibodies, which recognize cell surface molecules specifically or selectively expressed on tumor cells. Some targeting moieties, such as folate or transferrin, can enhance the cellular uptake of the NPs following their exit from leaky tumor vasculature [6,7]. However, targeting NPs with peptides or antibodies has limitations, including potential immunogenicity, relatively high cost, and lability in serum.

Aptamers (APs) represent an alternative form of NP targeting moiety [8]. An AP is a single-stranded structured RNA or DNA molecule that can bind to protein targets with affinities better than antibodies and afford better tumor penetration due to their smaller size [9]. APs which bind to specific protein targets are generated using a strategy known as systematic evolution of ligands by exponential enrichment (SELEX), in which libraries of random sequences are incubated with the target (here we focused on DNA APs, using a random ssDNA library), and DNAs that bind to the target are partitioned away from nonbinders and reamplified using PCR protocols, and the process is repeated until sequences with the desired binding affinities are identified. As examples, SELEX-generated APs against the prostate tumor marker prostate membrane-specific antigen have been used to safely and effectively direct chemotherapeutic drugs to prostate tumor cells [10,11], and epidermal growth factor (EGF)-receptor targeted APs conjugated to a gemcitabine-containing polymer inhibited *in vitro* proliferation of PDAC cells [12]. Therapeutic APs and AP-targeted delivery systems have moved into clinical trials [13,14].

The cell surface G protein coupled receptor *CCKBR* (Gene 1D#887) is overexpressed in many types of cancers and plays a role in tumor cell proliferation [15]. Natural *CCKBR* ligands gastrin and CCK, as well as *CCKBR* antagonists, have been attached to nanocarriers, radionuclides, and imaging agents to improve their uptake by tumor cells [16–23]. However, like most peptide targeting agents, the use of gastrin to direct cargo to tumor cells has been hampered by off-target binding, especially in the brain in the case of gastrin, as well as relatively high cost and serum instability/proteolytic degradation [4]. Moreover, gastrin can activate the *CCKBR* leading to proliferative signaling.

In this study, we describe selection and characterization of high-affinity DNA APs to the *CCKBR*. The initial AP chosen for study showed a K_d_ ∼15 pM, and we found that the APs are taken up by a *CCKBR*-mediated process and that they do not trigger *CCKBR* signaling or stimulate proliferation. Furthermore, bioconjugation to the surface of CPNSPs resulted in a major increase of uptake (even compared with gastrin) by orthotopic PDAC tumors *in vivo*.

## Materials and Methods

### Materials

Chemicals used were purchased as described: calcium chloride dihydrate (CaCl_2_·2H_2_O, ACS reagent, ≥99%), sodium phosphate dibasic (Na2HPO4, BioXtra, ≥99.0%), sodium metasilicate (Na2SiO3), sodium citrate tribasic dihydrate (HOC(COOH)(CH2COONa)2 · 2H_2_O, ACS reagent, ≥99.0%), N-(3-Dimethylaminopropyl)-N′-ethylcarbodiimide hydrochloride (EDC, commercial grade) from Sigma-Aldrich; indocyanine green (ICG) from TCI America; N-hydroxysulfosuccinimide (Sulfo-NHS) from Thermo Scientific; cyclohexane (ACS reagent, 99%+) from Alfa Aesar; methoxy (polyethylene glycol) amine hydrochloride salt (Methoxy-PEG-Amine, Mw 2 kDa), amine (polyethylene glycol) carboxyl hydrochloride salt (Carboxy-PEG-Amine, Mw 2 kDa), and maleimide (polyethylene glycol) amine trifluoroacetic acid salt (Maleimide-PEG-Amine, Mw 2 kDa) from JenKem Technology USA; polyoxyethylene [5] nonylphenylether (Igepal^®^ CO-520) from Rhodia.

PANC-1 and Cos-1 cells were obtained from ATCC. All cell lines were cultured in Dulbecco's modified Eagle medium with 10% fetal bovine serum and were periodically validated using ATCC kits, which use STR profiling (including Amelogenin). The COS-1 cell line, which does not express *CCKBR*, was used as a negative control in SELEX selections.

#### Systematic evolution of ligands by exponential enrichment

**FIG. 1.**
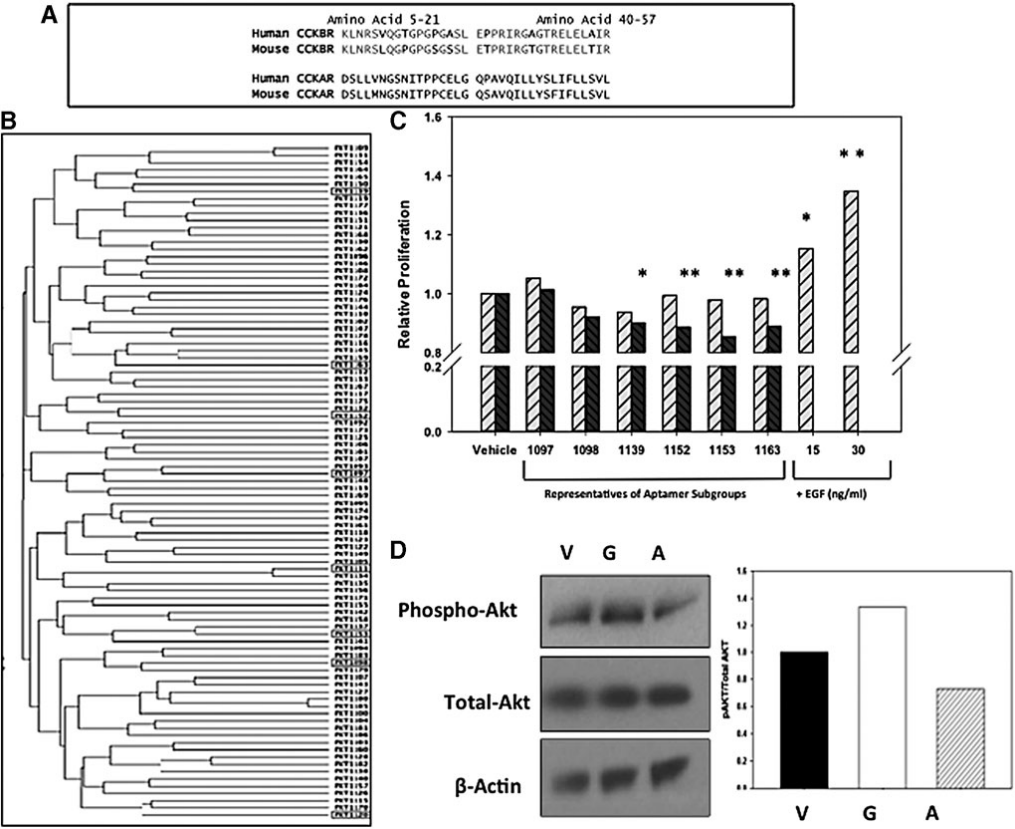
Selection of *CCKBR*-binding DNA aptamers. **(A)** Peptide sequences of the two regions of the *CCKBR* N-terminus (amino acids 5–21 and 40–57) used for initial aptamer selection and the sequence of the corresponding regions from the CCKAR. Residue in *bold* show amino acids which differ between human and mouse *CCKBR*. **(B)** Dendrogram comparison of the DNA sequence for selected *CCKBR* aptamers. Aptamers (*boxed*) were identified for further individual characterization. **(C)** Proliferation of PANC-1 human pancreatic cancer cells in the presence of *CCKBR*-selected aptamers or human recombinant EGF, a proproliferative control. Light cross-hatched bars represent 72-h time points, and darker cross-hatched bars represent 96-h treatment. Bars represent the standard error of the mean of three experimental replicates. None of the selected aptamers significantly stimulated pancreatic cancer cell proliferation compared to vehicle-treated cells. Instead, several APs produced significant growth inhibition by 96 h. *P < 0.05, **P < 0.01 compared to control (1152 and 1153 were significant at *P* < 0.001). **(D)** Western blots of vehicle (V), gastrin-17 (G), or AP1153 (A)-treated PANC-1 cells demonstrated that although gastrin-16 stimulates Akt phosphorylation, AP1153 did not. Adjacent panel shows densitometric quantification of the blot normalized for β-actin. AP, aptamer; CCKAR, cholecystokinin A receptor; *CCKBR*, cholecystokinin B receptor; EGF, epidermal growth factor.

Two peptides present in the N-terminal region of the human *CCKBR* (amino acids 5–21; BR5–21 and amino acids 40–57; BR40–57; Fig. 1) were synthesized with an added C-terminal cysteine residue to permit covalent coupling of the peptide to glass beads (GenScript). Coupling was achieved using aminosilane and sulfo-SMCC crosslinking reagents (Pierce), using 5 μg peptide and 200 mg glass beads, for 3 h with periodic mixing at room temperature.

A random 45 nucleotide library (45 nt library, N45) with fixed 5′ and 3′-flanking sequences was obtained (Integrated DNA Technologies). The fixed 5′-flanking sequence was 5′-CGCTCTAGAGTCGAATCA-, and the fixed 3′-flanking sequence was -GCGGCCGCTAATCCTGTT-3′. Following gel purification, the random N45 library with attached 3′-flanking sequence (at 500 nM) was mixed with *CCKBR* peptide-conjugated beads. After incubation for 60 min at 37°C, DNA APs bound to the peptide were harvested [24]. APs were reamplified through our PCR-based protocol, and this binding screen was repeated for eight rounds. The selected *CCKBR* peptide-bound “first-generation” AP pools were then cloned and sequenced. Two selection protocols were done in parallel, one using the *CCKBR* peptide BR5–21 and the other using the BR40–57 peptide, to identify two pools of *CCKBR*-specific APs.

Peptide-based AP selection was then coupled with a cell-based selection to ensure that the APs bound to *CCKBR* in its native cellular conformation. PANC-1 human PDAC cells were used for positive AP selection, and COS-1 cells, which do not express *CCKBR*, were used as a negative selection step for each round. Cells were grown to ∼80% confluency on 30 mm plates (∼10^6^ cells). Cells were rinsed twice in phosphate-buffered saline (PBS) and once in SB, and then incubated with the selected library pools (250 nM in 1.4 mL) and incubated for 30 min at 37°C.

Following incubation, cells were thoroughly rinsed with SB and harvested in 1 mL of 0.05% trypsin-EDTA solution. Additional rounds of selection that combined both peptide and cell-based selection were performed as glass beads only (background); glass bead with specific BR peptide; COS-1 cells (negative for *CCKBR*); and PANC-1 cells (positive for *CCKBR*). For unknown reasons, we were not able to use our minimal-primer protocol with the cell-based selections without modification. We therefore performed the selections with the 17 nt 3′-fixed primer remaining attached to the random 45mer region. After 22 rounds of positive and negative selection, clones from this final pool were sequenced and aligned using Clustal W2.1 (EMBL-EBI website).

Secondary structures were modeled using the Mfold program (http://mfold.rna.albany.edu), and eight prototype APs with stable predicted secondary structures and low free energies were chosen.

### AP characterization

AP dissociation constants against the *CCKBR* selection peptide were determined using APs that were labeled at the 5′-end with [γ-^32^P] ATP. The dissociation constant (K_D_) was determined by incubating various concentrations of AP (1–16,000 pM) with *CCKBR* peptide-conjugated glass beads (5 μg peptide). Unbound complexes were removed by washing, and bound APs were then eluted from the beads. Radioactivity in the peptide-bound fraction was determined by scintillation counting. Our standard control was unconjugated glass beads (which were equivalent to beads coupled to an irrelevant AP), which was subtracted (and minimal).

### Cell proliferation assays

PANC-1 cells were seeded (5,000 cells/well) into a 96-well plate and grown overnight. After 24 h, cells were transferred to fresh media containing 1% bovine serum and either 100 nM of each AP or an equal volume of the PBS vehicle was added. Treatment with human recombinant EGF (15 and 30 ng/mL) served as a positive control for stimulation of cell proliferation. Following a 72- and 96-h incubation, alamar Blue reagent (Life Technologies) was also added and absorbance was measured at 570 nm with a reference wavelength measured at 600 nm.

### AP uptake by three-dimensional confocal microscopy

For cell-based AP uptake studies, wild-type PANC-1 cells (WT), as well as two PANC-1 sublines, previously described in [25], were used. The first PANC-1 subline overexpresses the human *CCKBR* (subline *CCKBR* OE, which expresses the *CCKBR* at ∼2–3 × WT), and a second PANC-1 subline has reduced *CCKBR* expression due to knockdown by stable shRNA transfection (*CCKBR* KO; the reduction in *CCKBR* mRNA is 90%). *CCKBR* expression in the OE and KO sublines, and in WT PANC-1, was verified by qRT-PCR before use. Briefly, RNA was extracted using an RNAeasy kit (Qiagen, Valencia, CA), and cDNA was produced using a cDNA Reverse Transcriptase Kit (ABI, Carlsbad, CA) from 2 μg of RNA. Real-time PCR utilized 200 ng of cDNA per reaction, ABI Taqman master mix, and human CCK-BR primers (HS00176123_m1), with cyclophilin A (HS99999904_m1) as the internal standard.

For confocal microscopy, all three cell lines (WT, *CCKBR*-OE, and *CCKBR*-KO) were grown to near confluency on glass coverslips. Following incubation in serum-free media, cells (roughly 10^4^ cells) were treated with 10 nM of AlexaFluor488-conjugated APs for up to 24 h. AP1153, which was positively selected for binding to both *CCKBR* peptide and *CCKBR*-expressing cells, was compared to AP38, a first-generation AP that bound to *CCKBR* peptide but not to *CCKBR*-expressing cells, and to vehicle controls. After two PBS washes to remove unbound AP, cells were fixed in 2% paraformaldehyde for 30 min, washed twice in PBS and dH_2_O, and nuclei counterstained with Hoechst 33342 (0.5 μg/mL). Following brief rinses with PBS and dH_2_O, coverslips were mounted on slides with ProLong Gold (Life Technologies).

Images were acquired with a Leica AOBS SP8 laser scanning confocal microscope using a high-resolution Leica 40 × /1.3 Plan-Apochromat oil immersion objective. The three-dimensional (3D) stack images with optical section thickness (z-axis) of ∼0.3 μm were captured from cell volumes, z-section images were compiled, and final 3D image restoration was performed using VOLOCITY 6.3 software (PerkinElmer). The computation of AP voxel intensities was performed on the 3D image data sets recorded from at least three different areas of each cell line: OE PANC-1, WT PANC-1 wild-type cells (WT), and KO PANC-1 cells. A 2 × 2 kernel noise removal filter was used to remove the noise. Sum of all the voxel intensities above the lower threshold level was determined and was considered as the AP content. The same quantitation protocol was applied to all 3D image volume data sets generated from OE, WT, and KO samples and obtained using similar instrument setting parameters.

### Protein extraction and immunoblotting

Cellular proteins were extracted 24 h after treatment with *CCKBR* APs (100 nM). Protein concentration was determined using a MicroBCA assay (Pierce), and cell lysates (60 μg of protein) were resolved by SDS-PAGE. Proteins were transferred to nitrocellulose membranes, blocked in 5% BSA, and incubated overnight (4°C) with primary antibodies. Antibodies used were as follows: phosphorylated-Akt (Ser473) (#4060; Cell Signaling Technology), total Akt (#4691; Cell Signaling Technology), and beta-actin (#A2228; Sigma). The blots were washed and probed with secondary antibody coupled to horseradish peroxidase (HRP; Amersham), and HRP activity was detected using an enhanced chemiluminescent substrate (Pierce).

### Synthesis of AP-coupled NPs

Spherical CPSNPs doped with ICG were synthesized using aqueous precipitation of calcium chloride and disodium hydrogen phosphate in the presence of disodium silicate as described [25]. ICG doping was accomplished through the addition of the fluorophore into the microemulsion phase such that the ICG molecules were trapped and internalized within the particle. CPSNPs were laundered using van der Waals high-performance liquid chromatography. The fluorophore encapsulation yield was determined by comparing the concentration of ICG encapsulated within the CPSNPs to the initial concentration of the fluorophore added. After the particles were dissolved to release the dye, ICG content was quantified by the optical absorption at 785 nm and compared to a standard curve.

A 3′-NH2-TTTTT version of the *CCKBR* AP1153 (1 μM final concentration, TriLink) was covalently coupled to CPSNPs using an EDC/NHS coupling strategy as previously described [26]. The resulting AP-conjugated CPSNPs were dialyzed to separate unreacted APs and sterilized by filtration through a 0.2 μm cellulose membrane. To substantiate the surface functionalization of CPSNPs, zeta potential distributions were collected with a Brookhaven ZetaPALS zeta potential analyzer (Brookhaven Instruments Corp.) using Zeta PLUS mode. The samples were prepared with a dilution of 1:5 in pH-adjusted 70/30 ethanol/H_2_O (V/V). Four replicate measurements (five data points/run) were conducted to calculate the average zeta potential and 95% confidence interval.

To conduct particle size analysis, a drop of the 70/30 ethanol–water CPSNP suspension was diluted 1:3 and transferred onto a copper transmission electron microscopy (TEM) grid for imaging at 120 kV on the FEI Tecnai G2 Spirit BioTWIN TEM (Materials Characterization Laboratory, Pennsylvania State University). Images were processed on Image J (NIH), and the size histogram (*n* = 300) was generated and analyzed with the Gaussian multipeak function on Origin (OriginLab).

### *In vivo* tumor imaging

All animal procedures were approved by the Penn State Hershey Institutional Animal Care and Use Committee. Orthotopic PDAC xenografts were established by injecting 5 × 10^6^ PANC-1 cells in a 50 μL volume into the pancreas of athymic male nude (*nu/nu*) mice (Charles River). Orthotopic tumors were grown for 4 weeks before imaging. ICG-loaded CPSNPs, including nontargeted CPSNPs and CPSNPs bioconjugated with either gastrin 16 (G16) peptide or the AP1153, or empty (non-ICG containing) CPSNPs were resuspended in sterile 1 × DPBS (without Ca or Mg; MediaTech). Each mouse received a single ICG dose of 30 μg/kg, in a 100 μL volume, injected into the tail vein (*n* = 4 mice/group). At 18 h postinjection, mice were sedated and whole animal imaging was performed. Near-infrared transillumination images (755 nm excitation, 830 nm emission) and corresponding X-ray images were obtained with an *In vivo* FX whole animal imager (Carestream Health). Signal distribution relative to anatomy was illustrated by merging false-colored near-infrared and X-ray images.

To assess cell uptake *ex vivo*, the experiment was repeated, mice were sacrificed, and they were perfused with cold PBS (pH 7.4) containing 100 U/mL heparin at a rate of 1 mL/min until the perfusate was clear. Tissues were harvested and fixed in 4% paraformaldehyde, embedded in OCT medium, and stored at −20°C. Whole tumor cross sections were imaged using Nikon A1 MP+ Multi-Photon Microscope system (Nikon Instruments, New York) to assess localization of ICG relative to fibrotic regions of the tumor as well as the fibrillar collagens by second harmonic generation (SHG). A femtosecond IR laser source can induce harmonic generation signals from fibrillar collagens [27,28], enabling direct visualization of fibrosis without the use of exogenous probes, histological sectioning, or staining.

The laser used for SHG as well as the fluorescence emission from ICG was a mode-locked, femto-second, Spectra-Physics InSight DS femtosecond single-box laser system with automated dispersion compensation tunable between 680 and 1,300 nm (Spectra-Physics, Mountain View, CA). The laser output was attenuated using AOTF, and the average power was consistently maintained below the damage threshold of the samples. The power-attenuated laser was directed to a Nikon scan head coupled with Nikon upright microscope system (Nikon Instruments). The laser beam was then focused on the specimen through a high numerical aperture, low magnification, long working distance, dipping objective, CFI75 Apo Water 25 × /1.1 LWD 2.0 mm WD specifically designed for deep tissue imaging and other *ex vivo*/*in vivo*/*in vitro* imaging. Upon entering the microscope, the laser beam was directed to the scanning mirrors and subsequently focused on the specimens. The backscattered emission from the sample was collected through the same objective lens. Nikon NIS Element Software was used for the image acquisition. Nondescanned detectors in the reflection geometry were used for capturing the 3D images. A high-sensitivity GaAsP detector was used for very efficient SHG signal collection, and a high sensitivity extended red photo multiplier tube was used for the far red channel detection of ICG signal.

For 3D image data set acquisition, the multiphoton excitation beam (tuned to 880 nm for SHG and tuned to 820 nm for ICG emission) was first focused at the maximum signal intensity focal position within the tissue sample and the appropriate detector levels (both the gain and offset levels) were then selected to obtain the pixel intensities within the range of 0–4,095 (12-bit images) using a color gradient function. Later on, the beginning and end of the 3D stack (ie, the top and the bottom optical sections) were set based on the signal level degradation. A series of 2D Images for a selected 3D stack volume were then acquired at scan speed, that is, 4 s per 1024 × 1024 pixels. The 3D stack images with optical section thickness (z-axis) of ∼1.0 μm were captured from tissue volumes. For each tissue volume reported here, z-section images were compiled and finally the 3D image restoration was performed using IMARIS (Bitplane, Switzerland).

### Statistical analysis

Results are expressed as mean ± standard error. Student's *t*-tests were used to evaluate statistical significance with a *P* < 0.05 considered to be statistically significant.

## Results

### Selection of human *CCKBR*-specific DNA APs

Using an iterative SELEX approach, a pool of high-affinity DNA APs, which recognizes and binds to N-terminal extracellular regions of human *CCKBR*, was identified. The *CCKBR* peptides against which the AP selection was applied were chosen based on three criteria: the peptide was (i) on an extracellular portion of the receptor, (ii) not in a region known to participate in receptor activation, based on previous functional studies [27,28], and (iii) without sequence similarity to the corresponding region of the related receptor, the cholecystokinin A receptor (CCKAR). Two peptide regions met these criteria: human *CCKBR* amino acids 5–21 and 40–57 (Fig. 1A). Both peptides are found on the extracellular portion of the N-terminus of the human *CCKBR* protein and participate in ligand recognition.

There was 76.5% amino acid identity between the human and mouse sequences for the 5–21 peptide and 83.3% identity between human and mouse for the 40–57 peptide (Fig. 1A). No conserved amino acid identity was noted between these two *CCKBR* peptides and the corresponding regions of the CCKAR from either human or mouse (Fig. 1A), suggesting that APs selected against these two peptides would not recognize CCKAR.

After eight initial rounds of AP selection, binding characteristics of the selected pools of APs disclosed K_D_'s of ∼100 pM and relatively stable Mfold-predicted secondary structures. However, these “first generation” APs showed relatively poor binding to PANC-1 cells, which express the *CCKBR* [29]. The SELEX protocol was therefore modified to include sequential peptide and cell-based selections, coupled with a negative–positive selection cycle strategy [30]. After 22 “rounds” of selection, the resulting pool of “second generation” APs was then cloned and sequenced (∼100). Clustal W alignment showed a family tree with three subfamilies (Fig. 1B). Interestingly, there was no strong sequence homology among APs to indicate a unique AP motif that would predict *CCKBR* binding. All APs were again modeled for secondary structure using the Mfold program [31]. Based on the Clustal alignment and predicted secondary structures, a panel of eight *CCKBR*-targeted APs (Fig. 1B, highlighted in red) were selected for further characterization. Four of the eight APs shared stretches of sequence similarity, comparable ΔG values, and areas of folded structure near the 3′ region that suggested they could adopt similar conformations.

### *CCKBR* APs do not stimulate PDAC cell growth

Activation of *CCKBR* signaling by CCK or gastrin requires interactions of the ligand with both the N-terminus of the receptor (including the AP target sites, residues 5–21 and 40–57) and several additional residues on the extracellular loops and transmembrane pocket of the receptor [32]. These complex receptor–ligand interactions result in a conformational change in the receptor that triggers the intracellular G-protein-coupled signaling cascade [33]. The *CCKBR* ligand gastrin, which is also highly expressed by PDAC cells, can stimulate growth of pancreatic, colon, and gastric cancer [34], and gastrin-stimulated PDAC cell growth can be blocked with a *CCKBR*-specific antagonist [35].

Since the selected DNA APs are quite different in structure from the native *CCKBR* ligands, it is unlikely that these APs could activate the receptor and induce intracellular signaling. However, to confirm this, cell proliferation assays were used to assess whether *CCKBR* AP binding affected growth of PANC-1 cells compared with a known PANC-1 growth factor, EGF. The selected APs were individually used to treat PDAC cells at concentrations of 100 nM, 100 times the concentration of gastrin required for optimal *CCKBR* activation. After 72 and 96 h of exposure, PANC-1 cell proliferation was not increased by any of the AP treatments, while both 15 and 30 ng/mL EGF stimulated PANC-1 growth (Fig. 1C). Several of the APs (i.e, 1098, 1152, 1153, and 1163) produced significant growth inhibition at 96 h (*P* values ranging from *P* < 0.05 to 0.001). In addition, when the individual APs were considered as a pool, significant growth inhibition was observed (*P* < 0.0008).

### AP1153 does not activate *CCKBR* receptor signaling

Because the *CCKBR*-selected DNA APs did not stimulate PDAC cell proliferation, it would follow that these APs do not activate this G-protein-coupled receptor. Confirmation that AP1153 did not activate the *CCKBR* signaling was done by assessing the phosphorylation state of Akt, a downstream signaling intermediate known to be associated with *CCKBR* activation [25]. PANC-1 cells were treated with AP1153, gastrin-16, or vehicle (Fig. 1D). While gastrin treatment increased Akt phosphorylation, indicative of *CCKBR* activation, a decrease in phospho-Akt was noted in the AP-treated cells or vehicle controls. Total Akt levels were unchanged by any of the treatments. Thus, unlike the native ligand gastrin, AP1153 binding does not activate *CCKBR*-associated signaling pathways and appears to reduce Akt signaling.

### *CCKBR* AP1153 characterization

**FIG. 2.**
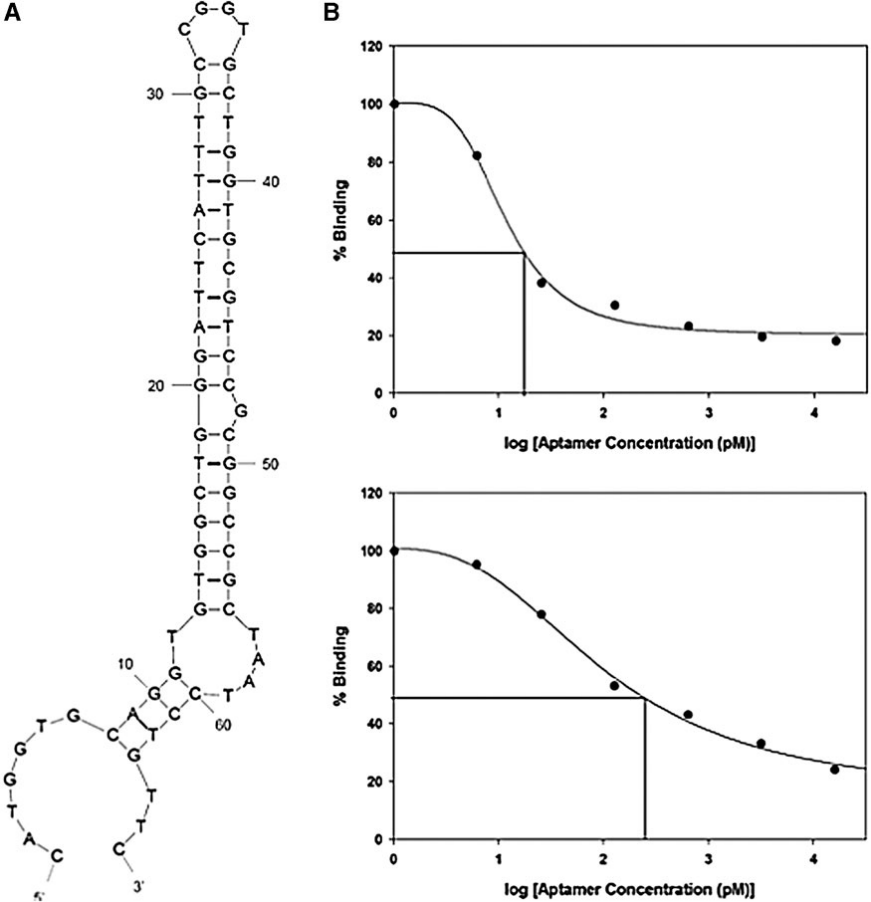
*CCKBR*-selected Aptamer 1153 Characterization. **(A)** Mfold predicted secondary structure of Aptamer 1153 (AP1153), which was selected as having the most stable secondary structure. **(B)** Dissociation constant (K_d_) determination for AP1153 against CCBR peptide. The 66-nucleotide version of the AP1153 (*upper panel*) and 49-nucleotide version of AP1153, which lack the 3′ region that was fixed in all aptamers in the original library (*lower panel*), had slightly different K_d_ values (15.5 vs. 206.2 pM). This is based on the assumption that x = y = 1 in the standard dissociation constant formula, so that K_d_ equals the concentration of free A at which half of the molecules of B are associated with A.

Of the eight APs chosen from the final pool, AP1153 had the most stable predicted secondary structure (Fig. 2A), with an estimated Tm of 54°C and a ΔG of −6.38 kcal/mol at 37°C. Dissociation constant (K_D_) measurements for AP against the BR5–21 peptide revealed a K_D_ of 15.5 pM (Fig. 2B). Since the K_D_ of gastrin for *CCKBR* is ∼1 nM, AP1153 has at least a 300-fold higher affinity for *CCKBR* than the native ligand. To further characterize this AP, the peptide-binding affinity of the full length, 66 nucleotide AP, was compared with a truncated version of AP1153 AP that lacked the nucleotides that are constant to the SELEX library vector. The truncated AP1153, 49 nucleotides in length, thus represented the DNA sequence unique to AP1153.

The K_D_ for the shorter version of AP1153 was 206.4 pM, and the secondary structure was predicted to be considerably less stable with a ΔG value of −1.94 kcal/mol (Fig. 2C). However, the K_D_ values for both APs were equal to or better than those recently reported for cell-SELEX-identified APs that recognize hepatocellular carcinoma cells [36], or an AP selected for binding to the EGF receptor [37]. Because of its higher affinity for the *CCKBR* and a more stable predicted secondary structure, subsequent experiments were done with the full-length, 66 nucleotide version of AP1153.

### *CCKBR* AP is internalized by a *CCKBR*-dependent process

To effectively use an AP to direct cargo to *CCKBR*-expressing PDAC cells, the AP should be taken up by receptor-mediated internalization. To demonstrate that AP1153 uptake was *CCKBR* mediated, internalization of fluorescent APs was assessed using wild-type (WT) PANC-1 cells, PANC-1 cells that have been engineered to constitutively overexpress the *CCKBR* (OE; these cells show increased expression of *CCKBR* of ∼2–3 × vs. WT), and PANC-1 cells that have been stably transfected with a *CCKBR* shRNA and have substantially reduced receptor expression (KO) [25].

**FIG. 3.**
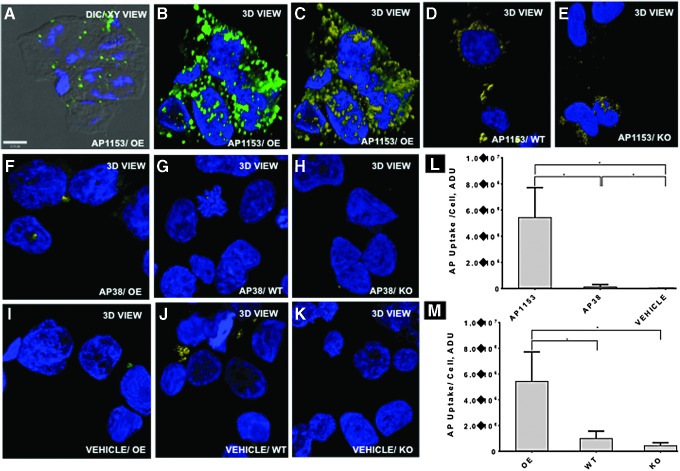
AP1153 uptake by PANC-1 cells is mediated through the *CCKBR*. Representative 3D confocal images showing AP1153 distributions in cultured PANC-1 cells that overexpress *CCKBR* **(A–C)**, PANC-1 wild-type cells **(D)**, and PANC-1 cells where the *CCKBR* has been knocked down by stable shRNA transfection **(E)**. The aptamer distributions are shown in *green*, while quantified voxels are shown *yellow*. *Blue* represents cell nuclei. Uptake of a first-generation aptamer, AP38, by PANC-1 cells overexpressing *CCKBR* **(F)**, wild-type **(G)**, and *CCKBR* knockdown cells **(H)** is significantly less than AP1153 **(L)**. All PANC-1 cells treated only with vehicle **(I–K)** show minimal background fluorescence. Integrated signal intensities from cell volumes **(M)** show that the *CCKBR* overexpressing cells take up significantly more AP1153 than either wild-type cells (WT) or *CCKBR* knockdown cells (KO) [*P < 0.05]. 3D, three dimensional.

AP1153 was readily taken up by PANC-1 cells that overexpress the *CCKBR* (Fig. 3A–C). Both DIC image and 3D image reconstructions confirm that AP1153 molecules do not simply remain at the cell surface, but are internalized and are present throughout the cytoplasm in multiple cells. To demonstrate that AP uptake was not a general nonspecific phenomenon, we also tested a first generation AP designated AP38; this AP bound to the isolated BR5–21 peptide but did not recognize the native receptor on PANC-1 cells. In the PANC-1 live-cell uptake experiments, AP38 was poorly taken up compared to AP1153 (Fig. 3F–H). Vehicle-treated cells showed little or no background fluorescence (Fig. 3I–K). Quantitation of cell-associated fluorescence confirmed that AP1153 internalization was markedly higher than internalization of AP38, and both were significantly higher than the background fluorescence in vehicle-treated cells (Fig. 3L).

As an additional control, PANC-1 KO cells that were stably transfected with a human *CCKBR* shRNA (resulting in a ∼80% reduction in *CCKBR* protein [25]) had even less cell-associated fluorescence (Fig. 3E and see Supplementary Fig S1 for *CCKBR* mRNA quantitation in the PANC-1 cell lines; Supplementary Data are available online at www.liebertpub.com/nat). The differences in AP1153 uptake by *CCKBR* OE, WT, and KO PANC-1 cell lines further indicate that AP internalization is *CCKBR* mediated (Fig. 3M). Finally, since PDAC cells secrete the *CCKBR* ligand gastrin, uptake of the *CCKBR* AP1153 by PDAC cells also indicates that binding and internalization of this AP by *CCKBR* can occur even in the presence of the native *CCKBR* ligand. Together, these data suggested that the AP1153 should effectively target and be taken up by PDAC cells *in vivo*.

### Bioconjugation of CPSNPs with a *CCKBR* AP or a gastrin G16 peptide

**FIG. 4.**
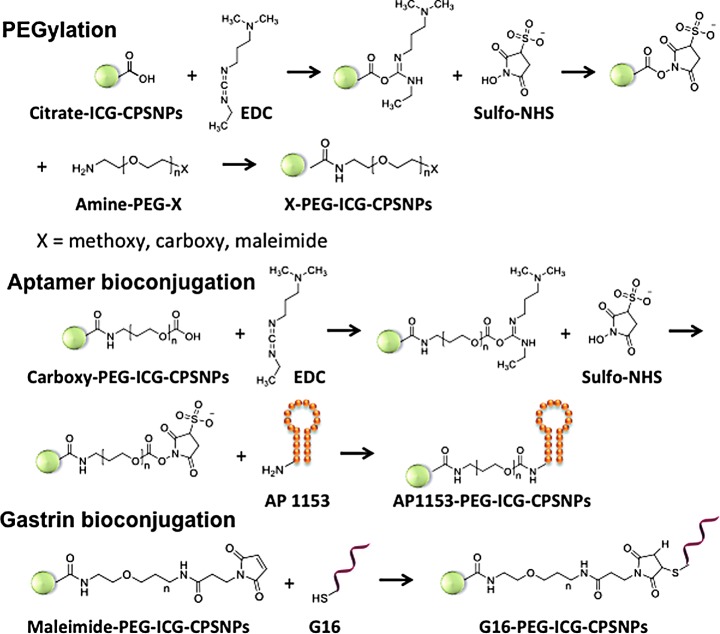
Bioconjugation strategies for targeted CPSNPs. A. Schematic of the steps used to bioconjugate either G16 peptide or AP1153 to the surface of CPSNPSs. After activation of the nanoparticle surface with PEG derivatives, functionalization with either AP1153 or G16 peptide was done as outlined. CPSNP, calcium phosphosilicate nanoparticle; G16, gastrin 16.

Using previously optimized methods for bioconjugation of targeting agents onto CPSNPs [23], two *CCKBR* targeting agents, G16 peptide and the AP1153, were attached to ICG-loaded CPSNPs (Fig. 4). ICG encapsulation efficiency, determined by comparing the amount of ICG released from CPSNPs to the initial fluorophore amount added during synthesis, was about 5 × 10^−6^ M, and the average fluorophore encapsulation efficiency was ∼0.8%. The fluorescence intensity of ICG-CPSNPs was at least five times of that of free ICG as a result of the matrix shielding effect of CPSNPs and multiple fluorophores encapsulated within each NP.

**FIG. 5.**
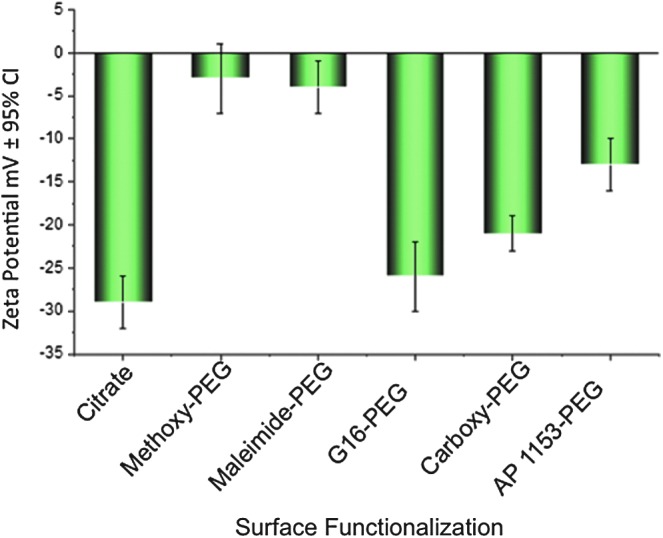
Zeta potential of CPSNPs synthesized with various surface functionalization. CPSNP surface functionalization with G16 peptide slightly decreases nanoparticle zeta potential, while bioconjugation with AP1153 results in a significantly less negative zeta potential. Error bars represent the 95% confidence interval of the mean of four independent measurements.

Because the surface of CPSNPs is altered by PEGylation and bioconjugation, overall CPSNP surface charge was determined by zeta potential analysis. Initially, CPSNPs displayed a negative average zeta potential value of −29 ± 3 mV at physiological pH due to the carboxyl groups from citrate on the particle surface (Fig. 5). After full surface coverage of CPSNPs with methoxy-PEG-Amine or maleimide-PEG-Amine, which have no net charge at pH 7, the zeta potentials shifted to −3 ± 4 and −4 ± 3 mV, respectively. Bioconjugation with G16 (Gastrin 16-PEG-ICG-CPSNPs) resulted in a more negative zeta potential of −21 ± 2 mV. In contrast, the initial zeta potential of carboxy-PEG-ICG-CPSNPs was −26 ± 4 mV, but shifted to −13 ± 3 mV after bioconjugation with AP1153 (Fig. 5).

Others have demonstrated that NPs with a slight negative charge have improved pharmacokinetic characteristics over particles with either a positive charge or highly negative charge [38,39]. In addition, NPs with protein decoration can have nonspecific interactions with serum proteins that mask targeting agents and eliminate selectivity [40]. Since AP1153-conjugated NPs are less negatively charged compared to G16 particles or nontargeted particles, the AP-modified particles should be less likely to aggregate with serum proteins *in vivo*. This property is likely to give AP-functionalized CPSNPs prolonged circulation time with less nonspecific cellular uptake and more effective delivery of their cargo to target cells.

**FIG. 6.**
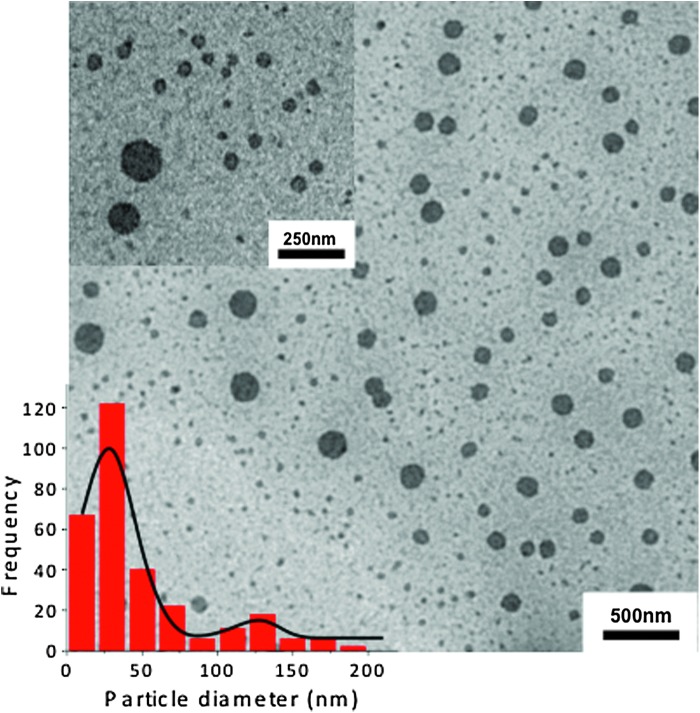
Transmission electron microscopy micrograph of AP1153-PEG-ICG-CPSNPS with *inset* highlighting the bimodal particle size distribution. The majority of CPSNPs are 30 ± 12 nm in diameter, whereas the tail distribution consists of larger particles (∼20% of the population), which are 121 ± 5 nm in diameter.

Finally, there was a bimodal particle size distribution of representative AP1153-PEG-ICG-CPSNPs (Fig. 6). The lognormal average diameter of ∼80% of the CPSNPs was 30 ± 12 nm, which is within the optimum particle size range for cellular uptake (Fig. 6, inset). Approximately 20% of the particles were larger, with an average diameter of 121 ± 5 nm.

### *In vivo* pancreatic tumor targeting with *CCKBR* AP

**FIG. 7.**
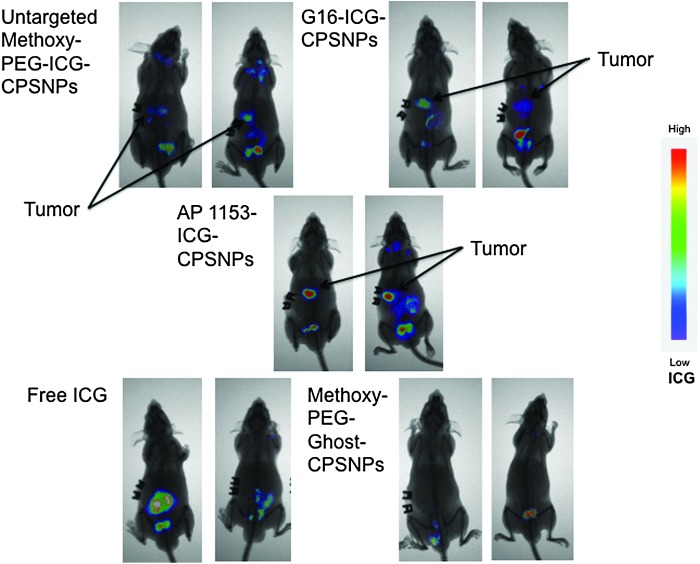
*CCKBR* aptamer enhances CPSNP uptake by PANC-1 Orthotopic Tumors *in vivo*. Athymic mice with established PANC-1 orthotopic tumors were treated with a single injection of ICG-loaded CPSNPs, bioconjugated with targeting agents as indicated, with unloaded, empty particles (Methoxy-PEG-ghost-CPSNPs), or with free ICG. ICG uptake by tumors was assessed by whole-body near-infrared imaging 15 h post-tail vein injection of particles. False color scale used to indicate ICG fluorescence intensity is shown at the *right*. AP1153 targeted particles become more concentrated in the orthotopic tumors than untargeted or G16-targeted particles. ICG, indocyanine green.

To directly compare the effect of targeting agents on NP uptake by PDAC tumors *in vivo*, CPSNPs surface bioconjugated with either G16 or AP1153 was injected into nude mice bearing orthotopic PANC-1 tumors. Peak tumor fluorescent was seen at ∼15–18 h after injection and was highest in the mice injected with AP1153-targeted NPs (Fig. 7). Background fluorescence in mice injected with empty particles was low and was mainly due to tissue autofluorescence in the GI and urinary tracts, or to the previously described enterohepatic biliary recirculation mechanism by which intact particles are cleared into the feces (Fig. 7) [41]. Free ICG, which is cleared through the biliary tree and gastrointestinal tract [42], showed no accumulation in the pancreatic tumors. Because ICG accumulation in tumors of mice treated with AP-targeted CPSNPs was higher than in tumors of mice treated either the untargeted or G16-targeted NPs, this suggests that the *CCKBR* AP targeted CPSNPs are taken up by pancreatic tumors more effectively.

**FIG. 8.**
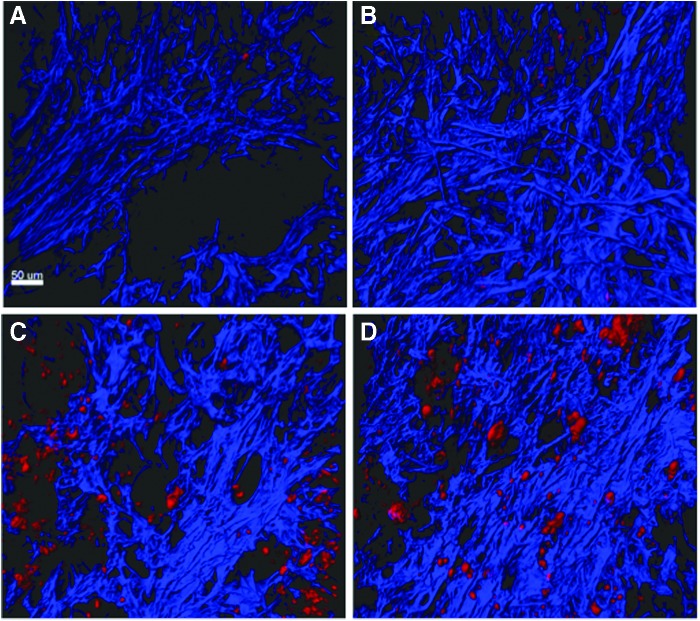
Enhanced delivery of AP1153-bioconjugated CPSNPs to Orthotopic PDACs. *Ex vivo* imaging of whole tumor cross sections with multiphoton microscopy demonstrated the location of ICG-loaded CPSNPs (false colored in *red*) relative to collagenous fibrotic regions of the tumor (*blue*). **(A)** Mice bearing tumors and injected with CPSNPs without ICG (ghost particles) showed little background fluorescence. **(B)** Tumors from mice injected with untargeted ICG-CPSNPs (with no aptamer bioconjugation) had some minimal tumoral uptake over background. **(C, D)** Tumors from two separate mice injected with AP1153-targeted ICG-CPSNPs showed increased ICG signal throughout the tumor sections. A 50 μm scale bar is shown in the lower left corner of panel **A**. PDAC, pancreatic ductal adenocarcinoma.

**FIG. 9.**
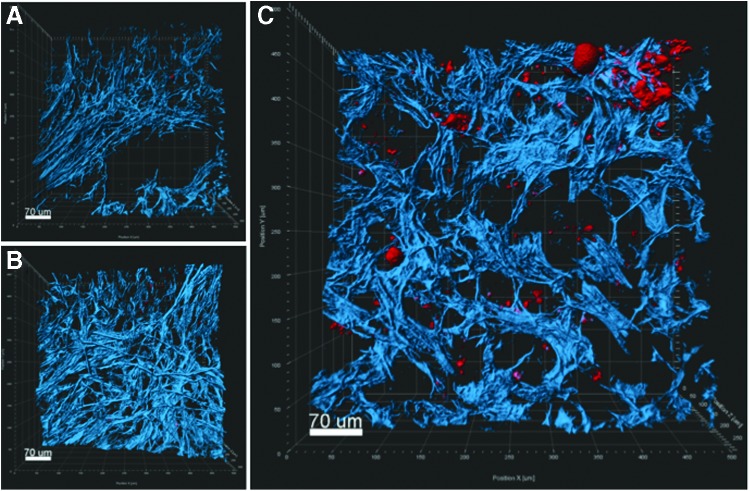
3D multiphoton images representing thick sections of orthotopic PDACs. Images show the spatial distributions of collagen (*blue*) along with ICG-loaded CPSNPs (*red*). **(A)** Ghost-CPSNPs; **(B)** untargeted-CPSNP; **(C)** AP-CPSNPs.

We also performed *ex vivo* imaging of the orthotopic pancreatic tumors using multiphoton and harmonic generation microscopy methods. The overall localizations of ICG-loaded particles were analogous to those observed in the *in vivo* imaging studies. *Ex vivo* imaging of cross sections of whole tumors showed that cellular uptake of the AP1153-targeted ICG-CPSNPs was clearly enhanced compared with untargeted ICG-loaded CPSNPs and was distributed throughout the cytoplasm (Fig. 8). The 3D reconstructions showed accumulation of ICG in tumor cells within the surrounding fibrotic stroma (Fig. 9).

## Discussion

Development of reagents that can direct chemotherapeutic drugs or imaging agents specifically to cancer cells is a critical need in the field of cancer biology. The selection and characterization DNA APs with high affinity for the *CCKBR*, a cell surface protein universally found on human PDAC cells, are a significant step toward that goal. Because APs were selected to conserved sites on the N-terminus of the *CCKBR*, which is extracellular, they can bind to the *CCKBR* and target CPSNPs to PDAC cells without activating proproliferative, antiapoptotic receptor signaling. Such tumor cell-specific targeting agents can assist in delivering cargos such as chemotherapeutics to pancreatic tumors with less uptake by normal cells, thereby reducing drug side effects and off-target toxicities. Because we have selected targeting APs that bind to the *CCKBR* with higher affinity than the native ligand, gastrin, the AP-conjugated CPSNPs should effectively out-compete gastrin for *CCKBR* binding, achieving cargo internalization even in the presence of gastrin secreted by the tumor (or stromal) cells.

The *in vivo* experiments herein demonstrated that *CCKBR*-AP-targeted CPSNPs were taken up by orthotopic PDAC tumors to a greater degree than were nontargeted or even gastrin-targeted CPSNPs. It is also important to note that CPSNPs bioconjugated with *CCKBR* APs did not demonstrate any uptake in the brain, the only adult tissue with high CCBR expression. Previous studies had shown that although gastrin-10 targeted CPSNPs were taken up by PDAC tumors, there was significant NP uptake in the brain [23]. AP-targeted CPSNPs do not appear to be present in brain tissues, possibly because they are not able to cross the blood–brain barrier. The lack of blood–brain barrier penetration is additional evidence that tumor targeting with a *CCKBR* AP offers significant advantages over gastrin targeting. In addition, it is possible that gastrin peptides on the NP surface could activate the *CCKBR*, stimulating the proliferation of tumor cells.

Since *CCKBR* APs do not activate receptor-associated signaling pathways, APs represent both a safer and more efficacious targeting agent for PDAC cells. These data are in agreement with another recent article, demonstrating that an anti-EpCAM AP was taken up *in vivo* by colon tumor xenografts more effectively than was an anti-EpCAM antibody [43].

The identification of an AP, which enhances the delivery of NPs to PDAC cells, could have multiple applications. First and foremost, they should allow early identification of pancreatic lesions. Since we have recently demonstrated that *CCKBR*s are also present on precursor PanIN lesions [44], these APs should improve early detection of PDAC tumors. Because surgery can be curative if lesions are detected early, the ability to identify precursor lesions before they progress to full-blown PDAC and metastasize could improve patient outcomes. Using tumor-targeted ICG-CPSNPs, near-infrared imaging during surgery could improve identification of tumor cells and permit surgeons to locate microscopic lesions and identify surgical margins in real time [42].

Currently, ICG is the only near infrared contrast dye that is FDA approved for clinical usage. By both encapsulating the ICG into CPSNPs and targeting the NPs for tumor-specific uptake, the amount of ICG required to visualize tumors in this study (30 μg/kg) was substantially less than the free ICG (up to 10 mg/kg) required to visualize subcutaneous murine tumors over an identical timeframe (up to 24 h postinjection) [42].

Codelivery of theranostic agents to both tumor and stellate cells, both of which express *CCKBR*, could further enhance treatment options for cancer patients. We anticipate that new *CCKBR* AP-targeted nanocarriers will have a broad capability to deliver imaging agents or therapeutic cargos specifically to PDAC cells with minimal off-target effects.

## Supplementary Material

Supplemental data
