# Supplementary material for: A Cholecystokinin B Receptor-Specific DNA Aptamer for Targeting Pancreatic Ductal Adenocarcinoma
Source: Nucleic Acid Ther. 2017 Feb 1;27(1):23–35. doi: 10.1089/nat.2016.0621 (PMC5312616; doi:10.1089/nat.2016.0621)
Supplement: Supplemental data [file Supp_Fig1.pdf]

Supplementary Data

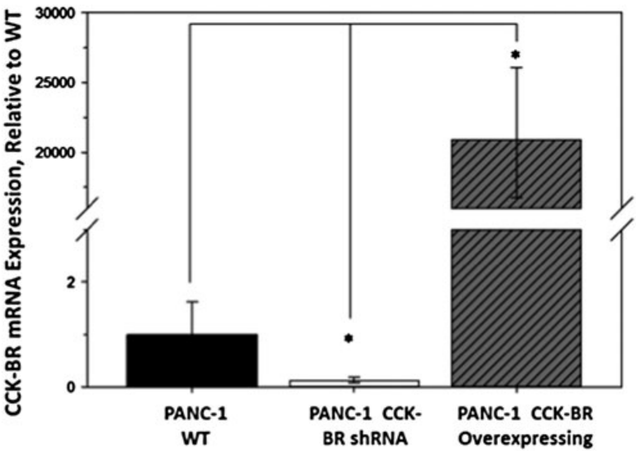

SUPPLEMENTARY FIG. S1. Quantification of *CCKBR* mRNA in PANC-1 clonal cell lines. Total RNA was isolated from PANC-1 wild-type cells (*solid bar*), PANC-1 cells where the *CCKBR* has been knocked down by stable shRNA transfection (*open bar*), and PANC-1 cells that overexpress *CCKBR* (*hatched bar*) and quantitated by qRT-PCR. Levels of *CCKBR* mRNA are expressed relative to WT PANC-1, and error bars represent the standard error of the mean of three independent RNA isolates. Student's *t*-tests determined that the amount of *CCKBR* mRNA was significantly different among all three lines (*$P < 0.001$). *CCKBR*, cholecystokinin B receptor.
